# GAMS5

**DOI:** 10.6028/jres.105.023

**Published:** 2000-02-01

**Authors:** C. Doll, H. G. Börner, T. von Egidy, H. Fujimoto, M. Jentschel, H. Lehmann

**Affiliations:** Physik-Department, Technische Universität München, D-85748 Garching, Germany; Institut Laue-Langevin, F-38042 Grenoble Cedex 9, France; Institut Laue-Langevin, F-38042 Grenoble Cedex 9, France; Physik-Department, Technische Universität München, D-85748 Garching, Germany; National Research Laboratory of Metrology, Tsukuba City, Ibaraki 305, Japan; Institut Laue-Langevin, F-38042 Grenoble Cedex 9, France

**Keywords:** crystal diffraction, gammaray spectrometer, nuclear spectroscopy

## Abstract

The construction of the double-crystal γ-spectrometer GAMS5 was finished recently and the instrument is now operational. Measurements with double flat crystals were carried out and we will report here on the progress concerning the characteristics of the spectrometer.

## 1. Introduction

**Fig. 1:**
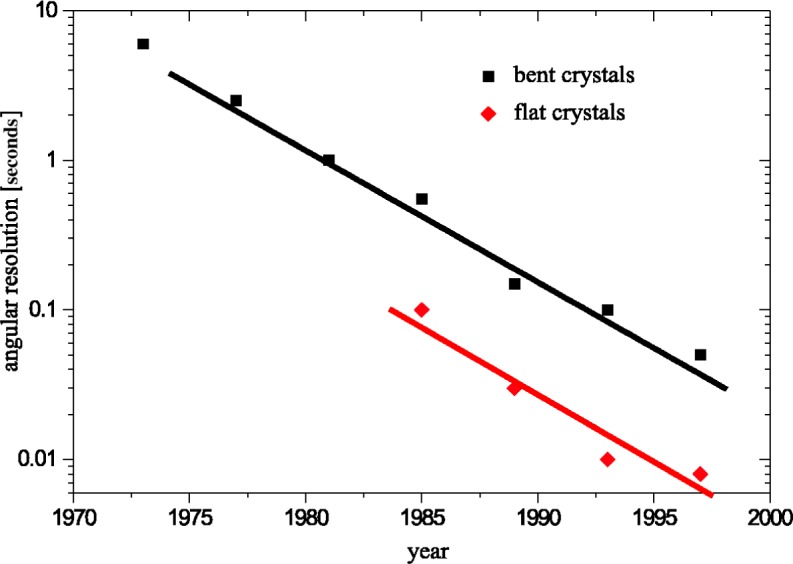
Development of the angular resolution on the Grenoble crystal-spectrometers over the last 25 years. The upper curve shows the progress on bent crystals whereas the lower curve represents flat crystals.

Neutron capture γ-ray spectroscopy studies at the High Flux Reactor of the Institut Laue-Langevin (ILL) in Grenoble were strongly influenced by the steady progress in development and improvement of crystal spectrometers. Wavelength-selection by Bragg-reflection on ideal single crystals allows to obtain to date a resolving power which is unequalled by any other γ-ray spectroscopic method. Figure 1 demonstrates that during the last 25 years the angular resolution obtained with ILL’s crystal spectrometers was steadily improved—overall by about three orders of magnitude. Whereas the very first measurements were carried out with bent crystals operated in DuMond geometry (several arcsec angular resolution at the very beginning) the best currently obtained resolving power $\left(\text{several}\;\text{marcsec}\;\equiv \frac{\Delta E}{E}\approx 10^{-6}\right)$ comes from measurements with flat crystals oriented in a two axis crystal geometry.

Two challenges emerged from this situation

- the centroid of a γ ray of energy *E* and width $\frac{\Delta E}{E}\approx 10^{-6}$ should be determined with a precision significantly better than that and
- the resolution obtained with bent crystals should come closer to that obtained with flat crystals.

The second item is especially interesting because the luminosity of a bent crystal mode is (due to the bigger effective solid angle) orders of magnitude higher than of a flat crystal mode (at comparable resolution). This challenge was addressed with the construction of a new crystal spectrometer—GAMS5—which is now in operation. We will, in the following, briefly discuss the current status of this spectrometer.

## 2. The GAMS5 Spectrometer

The two items to be improved i) the precision and ii) the resolution are—in first order approximation—independent issues. The first one concerns the spectrometer as a whole, the second one concerns crystals which might be interchanged on the spectrometer. Logically point i) had to be addressed first.

**Fig. 2:**
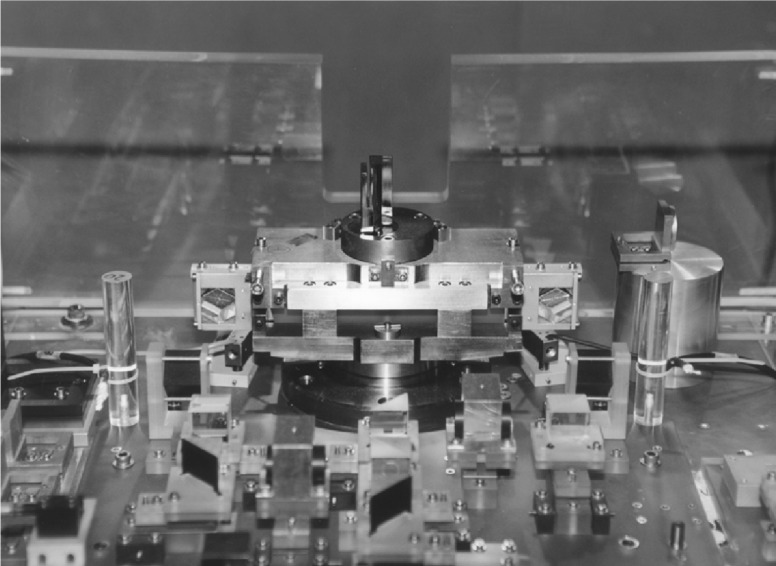
View of one of the interferometer-arms where a flat crystal is mounted.

In its basic conception the new spectrometer follows closely its predecessor at ILL, the GAMS4 spectrometer which was installed as the result of a longstanding collaboration between National Institute of Standards and Technology (NIST) and the Institut Laue-Langevin (ILL) [1] The γ rays are diffracted by a two axis crystal (either flat or bent) spectrometer used in transmission. Nearly ideal crystals (Si or Ge) are rigidly connected to the rotational axes whose angular rotations are measured with (see Fig. 2) Michelson type angle interferometers. The main difference to the GAMS4 spectrometer consists in the use of glass ceramics (ZERODUR instead of INVAR and/or iron) for all major building blocs of the interferometers. Due to the very low thermal expansion coefficient of ZERODUR ( $\frac{\Delta d}{d}\approx 5\times \frac{10^{-8}}{{}^{\circ}C^{-1}}$ at 20 °C) the effect of thermal gradients on the instrument are minimised. Nevertheless the temperature in the spectrometer housing is stabilised to better than 0.1 °C/d. Additionally, relevant temperatures are precisely measured and used for corrections. This concerns i) the air temperature to follow the laser wavelength, e.g., “length” of an interference fringe, ii) the crystal temperatures for the temperature dependence of the grating constant and iii) the interferometer arm to account for the interference fringe/angle ratio.

As the laser wavelength depends also on further environmental parameters like air pressure and humidity, these are also monitored and enter into corrections.

**Fig. 3:**
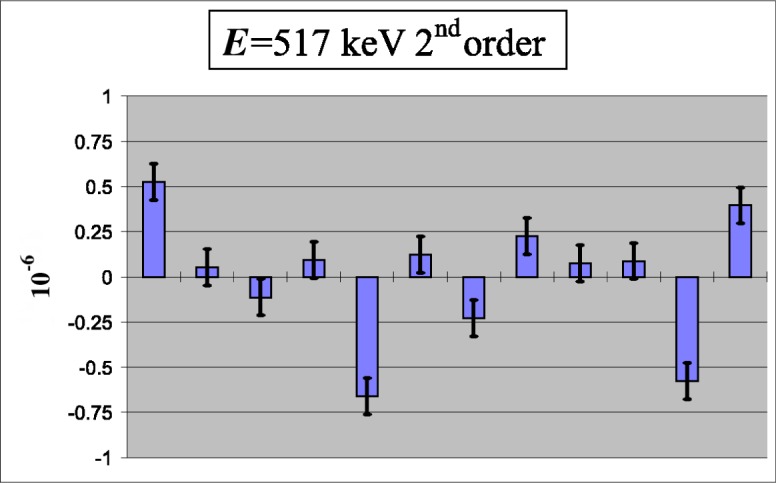
Scatter of the angular distances between the dispersive and nondispersive reflection of a 517 keV transition in ^36^Cl. This plot shows that within statistics the relative error is smaller than 2 × 10^−7^.

**Fig. 4:**
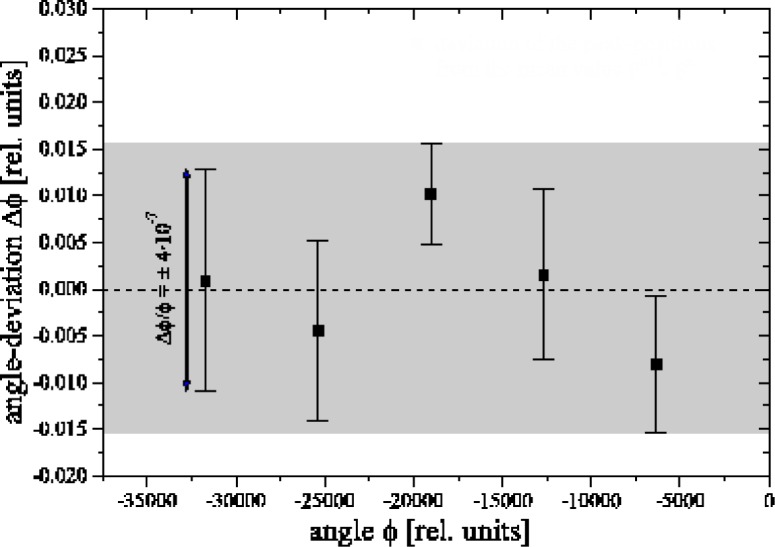
Measurement of the long-range (over ≈ 1°) nonlinearity. The plot shows the deviations from the average of the peak-positions for different reflection-orders plotted over the angle. Within statistics no deviations of the linearity are observed.

**Fig. 5:**
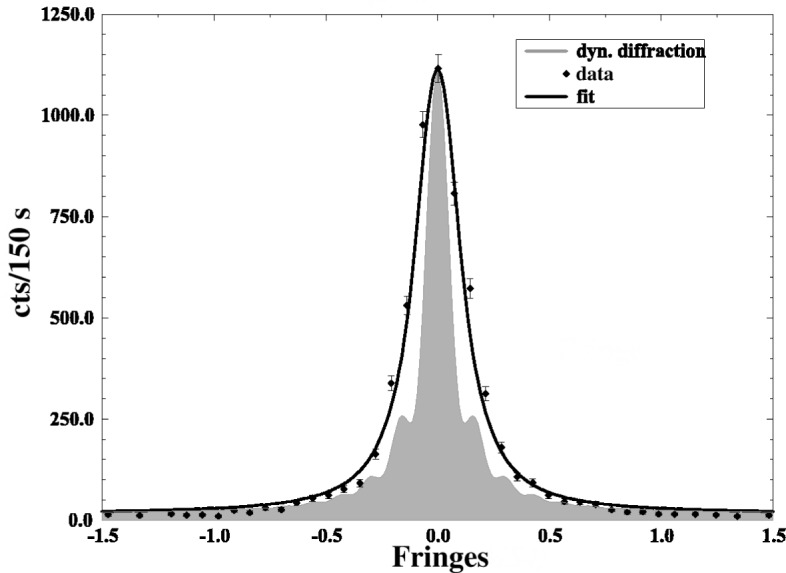
The instrumental resolution on the GAMS5 spectrometer, measured with flat, 2.7 mm thick Si crystals. The curve shown is for the 816 kev line in ^168^Er recorded using the Si (660) reflection. The shaded area shows the shape calculated from dynamical diffraction theory. The data points are fitted to a curve which is the dynamical diffraction theory folded with a gaussian (instrumental broadening) of 0.006″. This instrumental broadening of 0.006″ corresponds for this energy to an energy resolution of $\frac{\Delta E}{E}=1.2\times 10^{-6}$. On the abscess scale 1 fringe = 0.040″.

Measurements of reproducibility (Fig. 3) and linearity (Fig. 4) show that the spectrometer is robust on at least the 10^−7^ level and that the specifications are met. Meanwhile many important high resolution measurements have been carried out using flat crystals and the high inherent resolving power which can be obtained with these is shown in Fig. 5. Consequently the first part of the new development programme can be considered as being completed successfully.

**Fig. 6:**
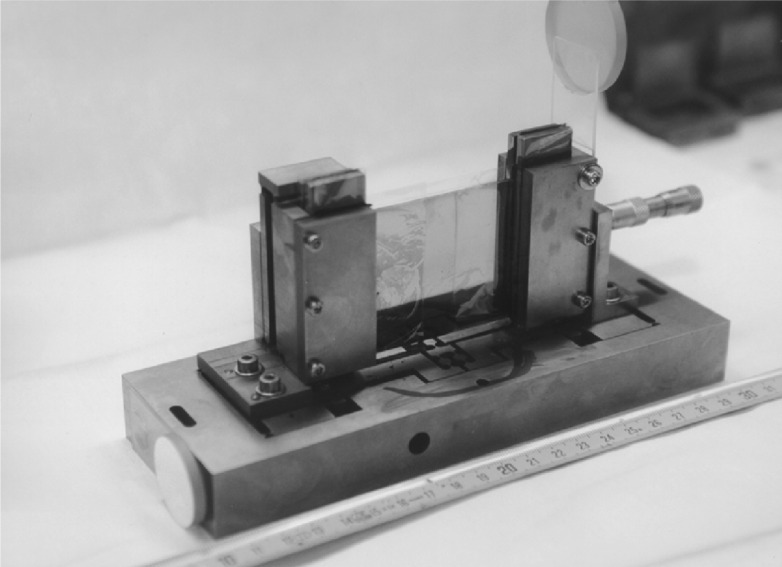
View of one of the devices which are used to bend the crystals.

The development of new more performant bent crystals is currently in progress. Two new Si-crystals are now available and first test measurements were started. Dynamical bending devices with incorporated flexure stages are used (see Fig. 6). Improvement on theses devices will proceed in parallel with measurements using flat crystals which are also open to ILL’s user community.
